# Supplementary material for: Mantle deformation records fossil convergent upwelling at Perm Anomaly
Source: Nat Commun. 2026 Apr 2;17:4746. doi: 10.1038/s41467-026-71070-2 (PMC13216635; doi:10.1038/s41467-026-71070-2)
Supplement: Supplementary file 1 — Supplementary Information [file 41467_2026_71070_MOESM1_ESM.pdf]

# Mantle deformation records fossil convergent upwelling at Perm Anomaly – Supplementary Material

**Jonathan Wolf[1,2,3,*], Mingming Li[4], and Barbara Romanowicz[1]**

[1]Department of Earth and Planetary Science, University of California, Berkeley, CA, USA

[2]Miller Institute for Basic Research in Science, Berkeley, CA, USA

[3]Department of Earth and Planetary Sciences, University of California, Santa Cruz, CA, USA

[4]School of Earth and Space Exploration, Arizona State University, Tempe, AZ, USA

[*]wolf@ucsc.edu

## Utilized Data

Data are publicly available were collected from the following on-line data centers: AUSPASS (https://auspass.edu.au/data.html), BGR (https://eida.bgr.de/), CNDC (https://www.earthquakescanada.nrcan.gc.ca/stndon/CNDC/index-en.php), Earthscope (http://service.iris.edu/), ETH (https://eida.ethz.ch/), FNET (https://www.fnet.bosai.go.jp/top.php?LANG=en), GEOFON (https://geofon.gfz-potsdam.de/)[1], GDMS (https://gdmsn.cwb.gov.tw/)[2], ICGC (https://www.icgc.cat/en/Ciutada/Explora-Catalunya/Terratremols), INGV (http://cnt.rm.ingv.it/en/webservices_and_software), IPGP (http://ws.ipgp.fr/)[3], KNMI (http://rdsa.knmi.nl/), KOERI (http://www.koeri.boun.edu.tr/new/en), LMU (http://erde.geophysik.uni-muenchen.de/), NCEDC (https://ncedc.org/)[4], NIEP (https://www.infp.ro/), NOA (http://bbnet.gein.noa.gr/HL/), OHPDMC (http://ohpdmc.eri.u-tokyo.ac.jp/), ORFEUS (http://www.orfeus-eu.org/), RESIF (https://seismology.resif.fr/)[5], SCEDC (https://scedc.caltech.edu/)[6], SSN (http://www.ssn.unam.mx/)[7], TEXNET (http://rtserve.beg.utexas.edu/), and USP (https://sismo.iag.usp.br/). All networks and network citations are included as additional Supplementary Materials, and were derived from the FDSN network code list (https://fdsn.org/networks/).

## Seismic tomography models used in the cluster analysis by Lekic et al. (2012)

SAW24B16[8], Houser et al. (2008)[9], S362ANI[10], GyPSuM[11], S40RTS[12]

## Supplementary Figures

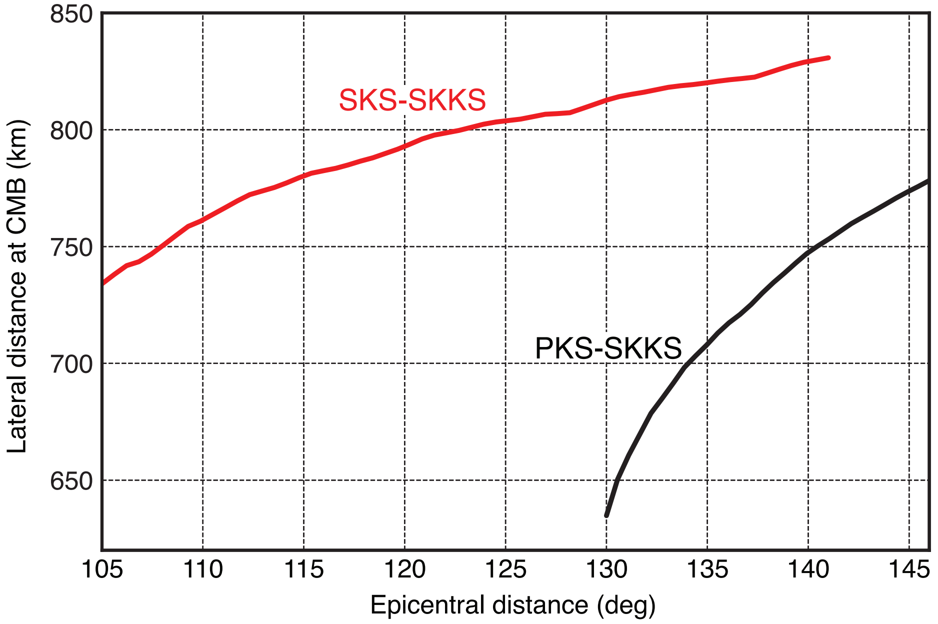


**Figure S1.** Lateral distance for SKS-SKKS (red) and PKS-SKKS (black) near the core-mantle boundary as a function of epicentral distance, for the epicentral distances used in this study.

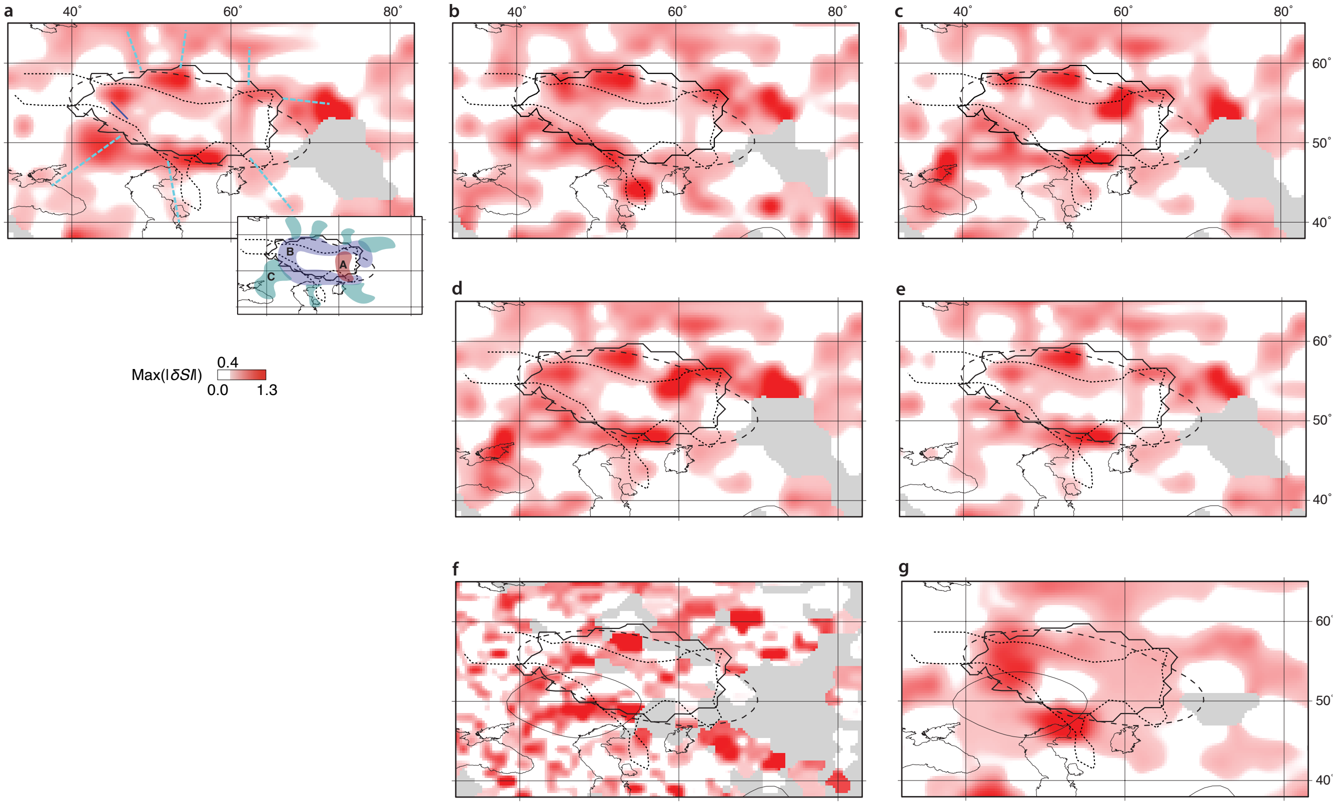


**Figure S2.** Results obtained using various averaging schemes. **a** Like Figure 2 in the main manuscript, averaging 30°-wide backazimuthal swaths, starting from 0°. Background colors show $Max(|\delta SI|)$ values (legend). Gray indicates no ray coverage. Black lines show locations of the Perm Anomaly determined by a cluster analysis (solid line where majority of models show low velocities), tomography model GLAD-M25 (dashed line for S velocity outline at 7.23 $\frac{\text{km}}{\text{s}}$ at 2800 km), and by an S-ScS residual travel time analysis (dotted line at 0.7 % travel time anomaly). Linear anisotropic features are indicated by dashed light blue lines. Inset: Naming convention of anisotropic features referred to in the main manuscript. **b** Same as panel **a** but starting bins at backazimuths 10°, 40°, ... degrees. **c** Same as panel **a** but starting bins at backazimuths 20°, 50°, ... degrees. **d** Same as panel **a** but for 15°-wide backazimuthal swaths. **e** Same as panel **a** but for 45°-wide backazimuthal swaths. **f** Same as panel **a** but for 1 degree × 1 degree bins. **g** Same as panel **a** but for 4 degree × 4 degree bins.

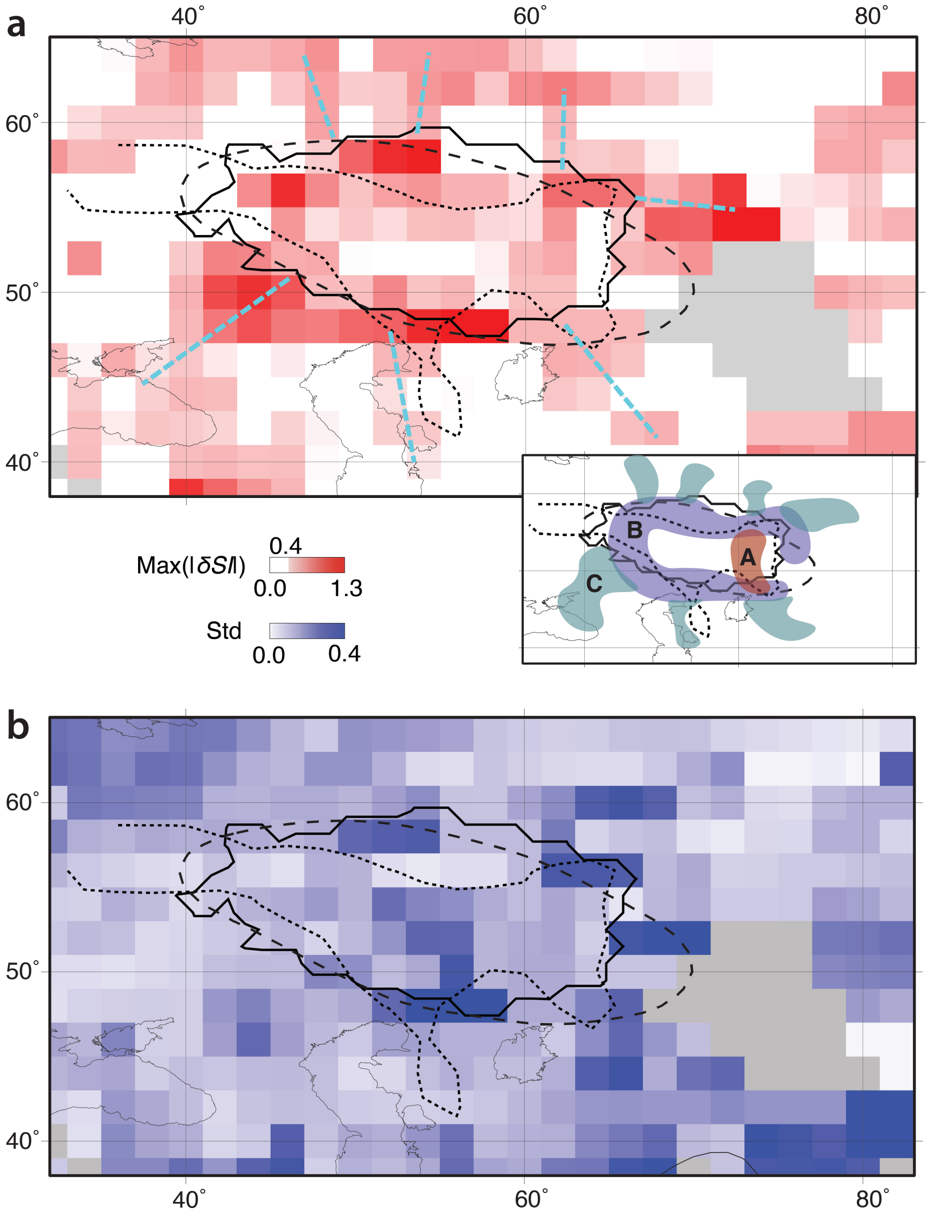


**Figure S3.** Result without smoothing and bootstrap uncertainties. **a** $Max(|\delta SI|)$ values (legend) as in Figure 2 of the main manuscript and Figure S2**a**, but without smoothing. **b** Uncertainties associated with $Max(|\delta SI|)$ values. The color scale (legend) represents bootstrap estimates of the uncertainty of the mean, computing the standard deviation of the distribution of sample means.

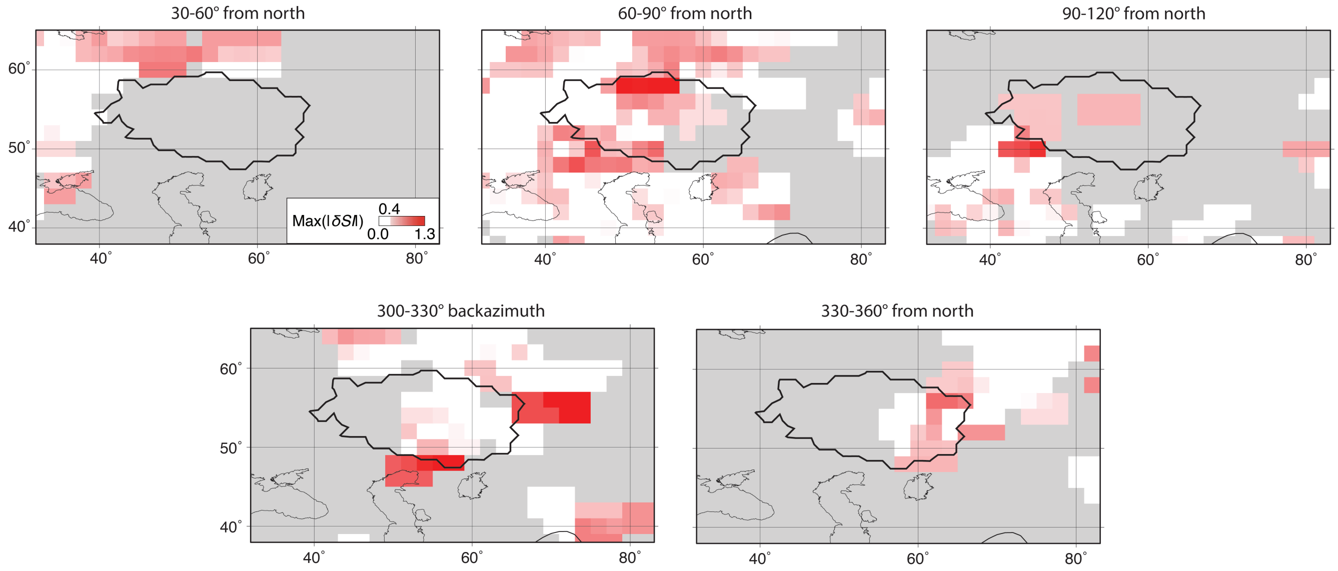


**Figure S4.** Splitting results for all five 30° directional intervals (header) for which we obtain measurements for more than three bins. Black lines show where a majority of models indicate low velocities in a cluster analysis. Background colors represent $|\delta SI|$ values (legend).

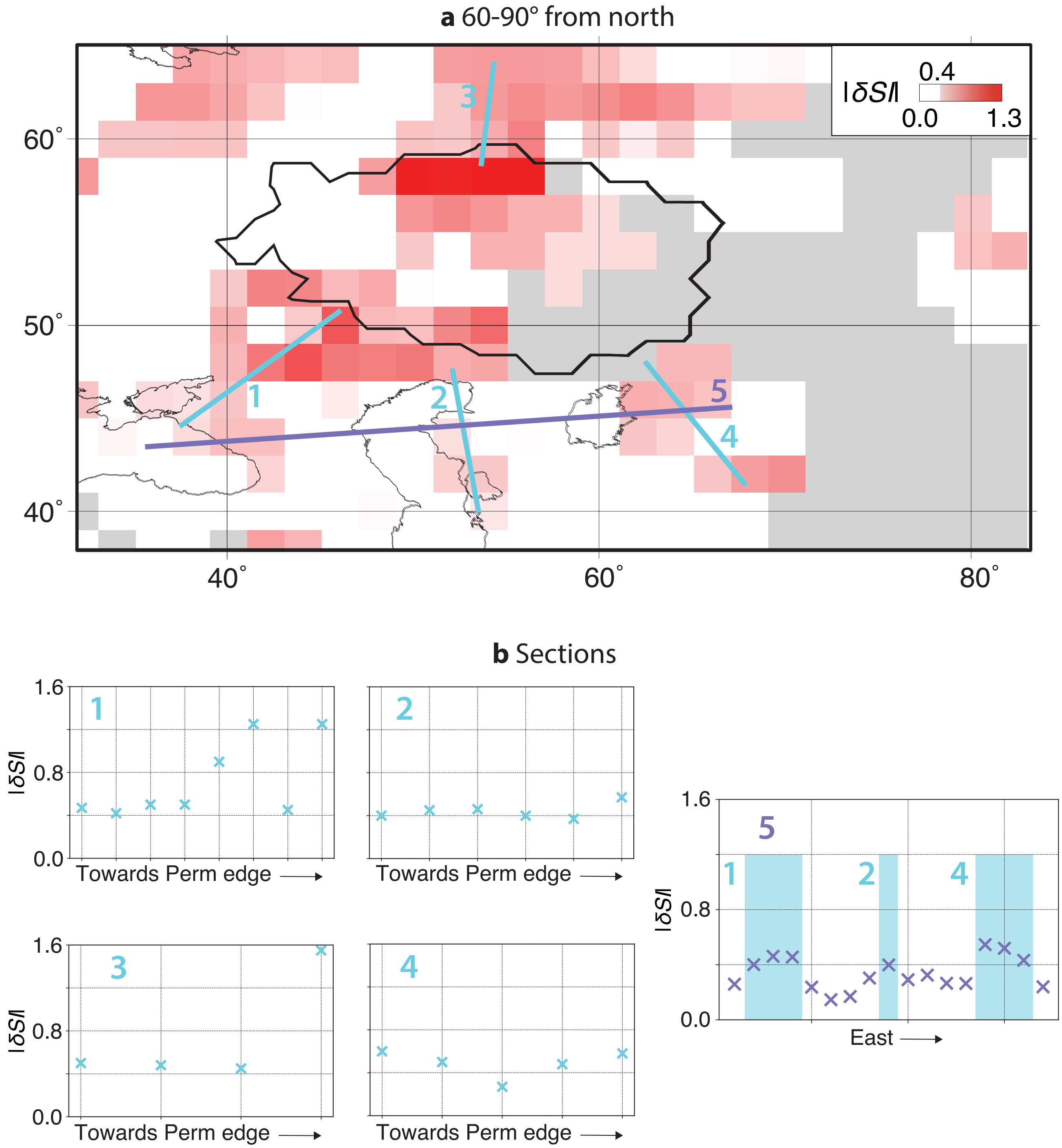


**Figure S5.** Results from the best sampled direction (60-90° from north). (a) Black lines show where three out of five models indicate low velocities in a cluster analysis. Background colors represent $|\delta SI|$ values (legend). Four linear anisotropic features (1-4) are marked in cyan. A section through three of the anisotropic features is labeled 5. (b) $|\delta SI|$ values along the four sections shown in panel (a).

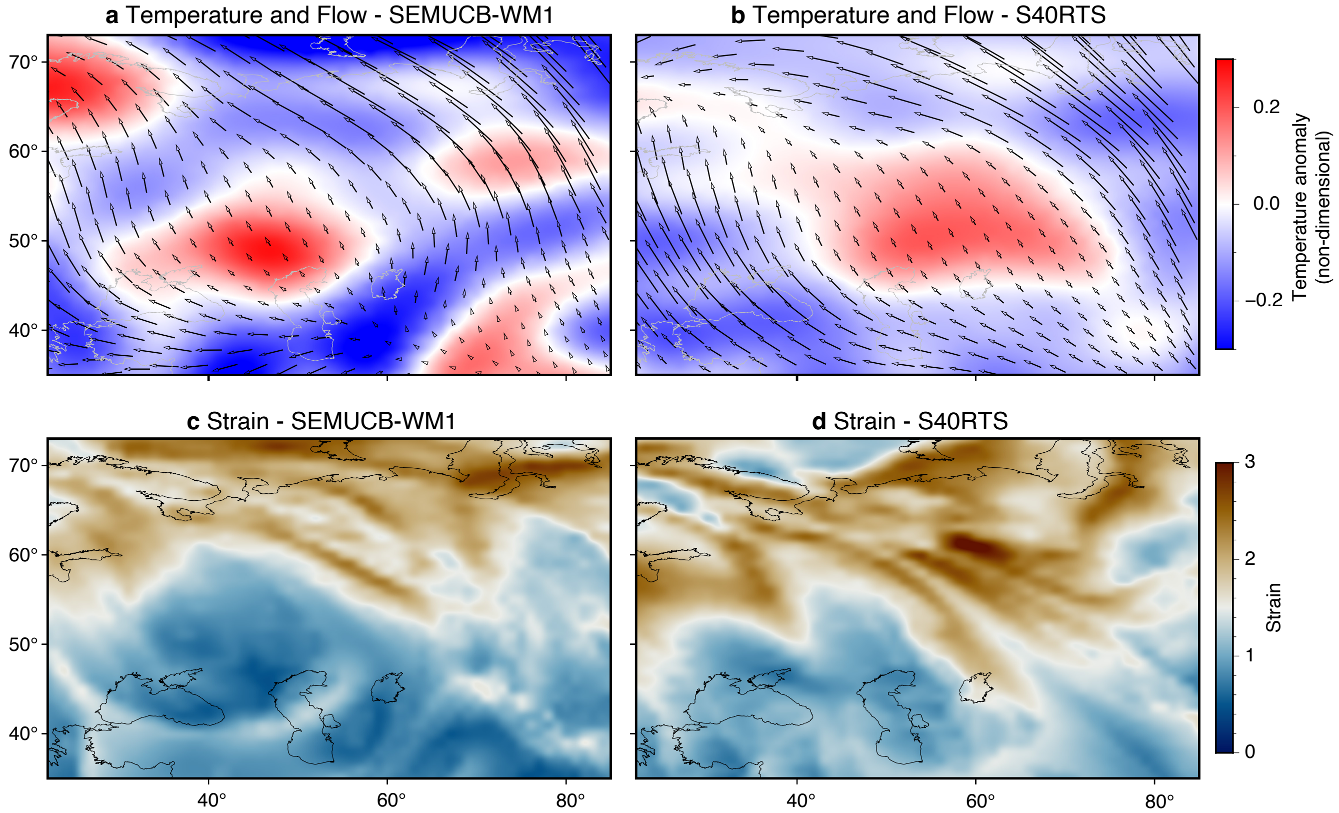


**Figure S6.** Geodynamic simulation following Li et al. (2024)[13] showing the present-day temperature anomaly, horizontal flow and strain 97.5 km above the CMB from seismic tomography models. **a** Non-dimensional temperature anomaly after the horizontal average is removed inferred from the tomography model SEMUCB-WM1. Black arrows indicate horizontal flow velocities. **b** Same as panel **a** for S40RTS. **c** Strain derived from inferred flow for SEMUCB-WM1. **d** Same as panel **c** for S40RTS.

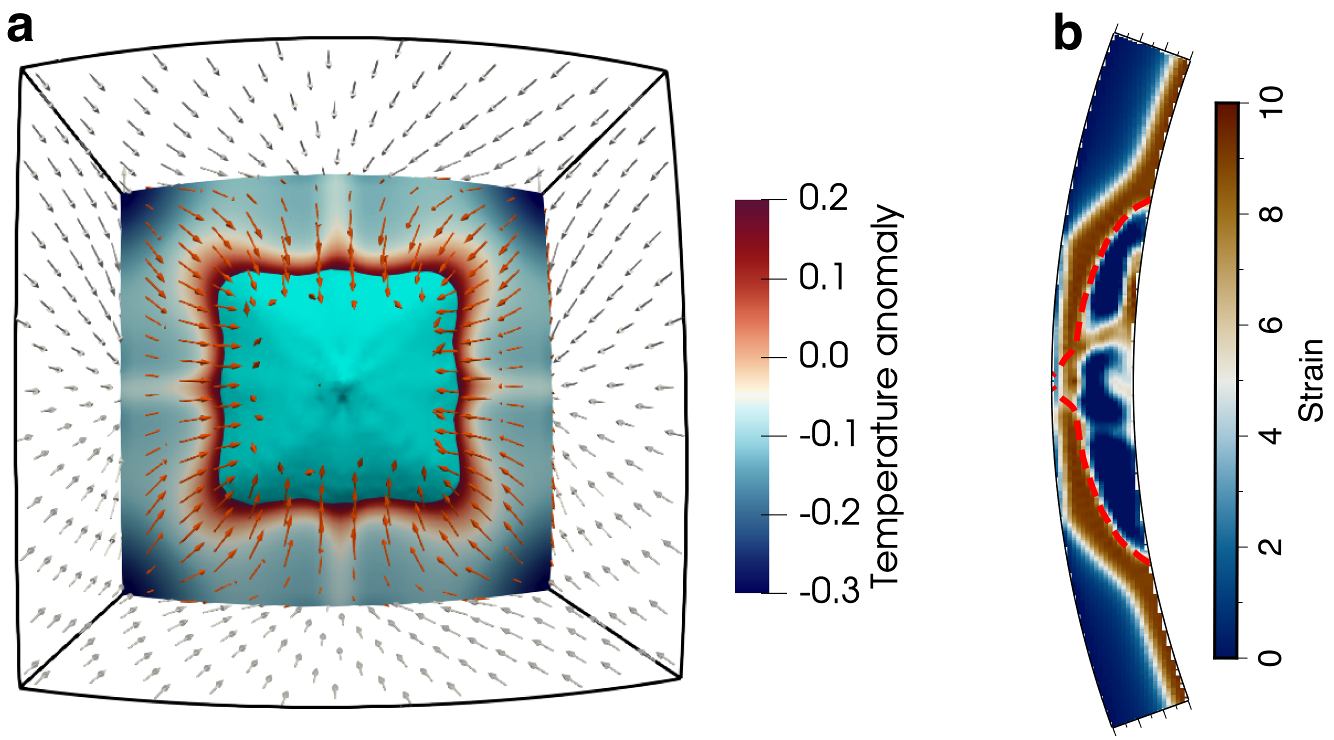


**Figure S7.** Snapshot of geodynamic modeling results. **a** Modeling results including flow velocity at the side boundaries (gray arrows), flow velocity (red arrows) and temperature anomaly (blue-to-red colors) 45 km above the CMB, and the thermochemical pile (cyan). Black lines show the model domain in the lower 1445 km of the mantle. **b** Vertical cross-section of strain along the equatorial regions in the lowermost 300 of the mantle. Red dashed line shows the pile edges.

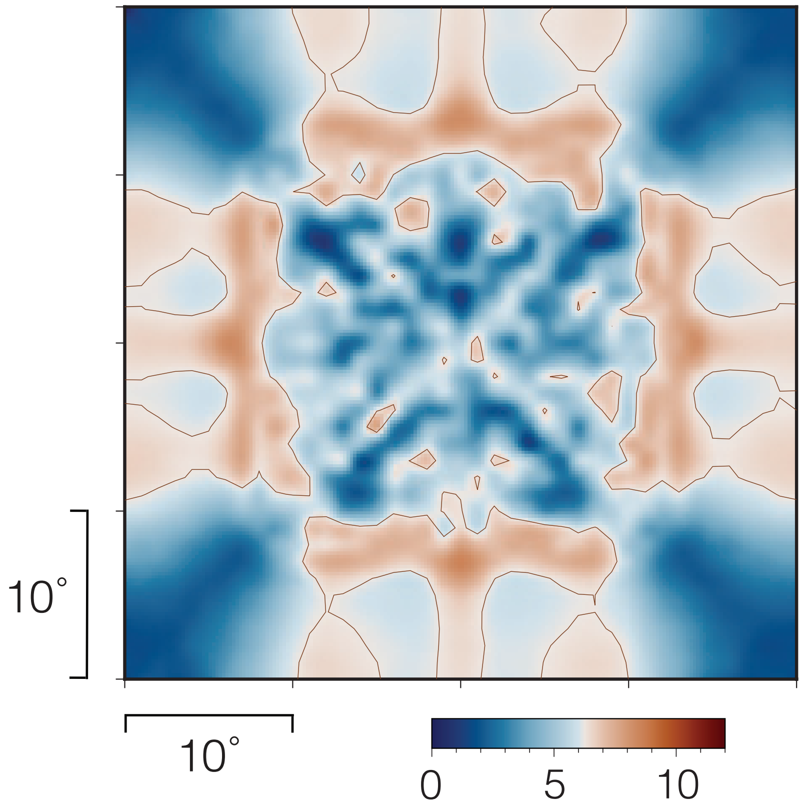


**Figure S8.** Geodynamically derived depth-averaged strain (legend, outline at 7.0) in the lowermost 67.5 km km of the mantle for a starting model with an initial 25 km (instead of 50 km) thick global layer of intrinsically dense material with a buoyancy number (see Online Method for definition) of 2.0 (instead of 0.8) is placed on the CMB.

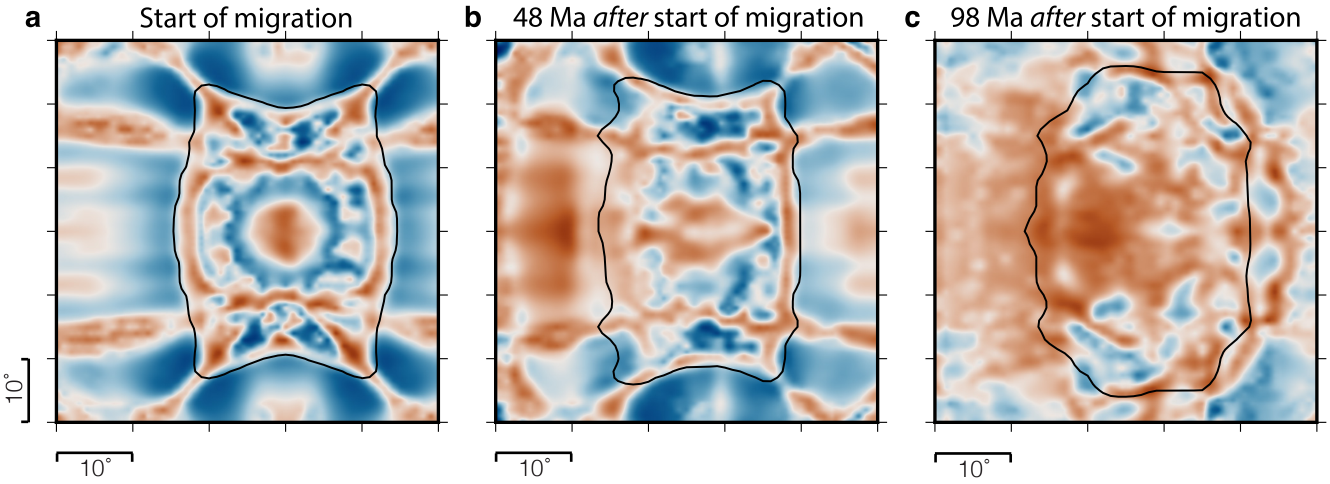


**Figure S9.** Evolution of depth-averaged strain in the lowermost 120 km of the mantle (see color scale) during the westward migration of the Perm Anomaly in a Type-2 geodynamic model. The panels show the system **(a)** at the onset of motion, **(b)** after 48 million years, and **(c)** 98 million years of drift. Views are restricted to the central model domain to exclude boundary artifacts. Solid black lines indicate the spatial extent of the thermochemical anomaly.

## References


1. GFZ Data Services. GEOFON Data Centre: GEOFON Seismic Network, DOI: 10.14470/TR560404 (1993).

2. (Taiwan), C. W. B. Central Weather Bureau Seismographic Network, DOI: 10.7914/SN/T5 (2012).

3. Institut de physique du globe de Paris (IPGP) & École et Observatoire des Sciences de la Terre de Strasbourg (EOST). Geoscope, french global network of broad band seismic stations, DOI: 10.18715/GEOSCOPE.G (1982).

4. UC Berkeley Seismological Laboratory. Northern California Earthquake Data Center, DOI: 10.7932/NCEDC (2014).

5. RESIF. RESIF-RLBP French Broad-band network, RESIF-RAP strong motion network and other seismic stations in metropolitan France, DOI: 10.15778/RESIF.FR (1995).

6. Caltech. Southern California Earthquake Center, DOI: 10.7909/C3WD3xH1 (2014).

7. Instituto de Geofísica, Universidad Nacional Autónoma de México, México. SSN: Servicio Sismológico Nacional, DOI: 10.21766/SSNMX/SN/MX (2024).

8. Mégnin, C. & Romanowicz, B. The three-dimensional shear velocity structure of the mantle from the inversion of body, surface and higher-mode waveforms. *Geophys. J. Int.* **143**, 709–728, DOI: 10.1046/j.1365-246X.2000.00298.x (2000).

9. Houser, C., Masters, G., Shearer, P. & Laske, G. Shear and compressional velocity models of the mantle from cluster analysis of long-period waveforms. *Geophys. J. Int.* **174**, 195–212, DOI: 10.1111/j.1365-246X.2008.03763.x (2008).

10. Kustowski, B., Ekström, G. & Dziewoński, A. M. Anisotropic shear-wave velocity structure of the earth's mantle: A global model. *J. Geophys. Res. Solid Earth* **113**, B06306, DOI: 10.1029/2007JB005169 (2008).

11. Simmons, N. A., Forte, A. M., Boschi, L. & Grand, S. P. GyPSuM: A joint tomographic model of mantle density and seismic wave speeds. *J. Geophys. Res. Solid Earth* **115**, DOI: 10.1029/2010JB007631 (2010).

12. Ritsema, J., Deuss, A., van Heijst, H. J. & Woodhouse, J. H. S40RTS: a degree-40 shear-velocity model for the mantle from new Rayleigh wave dispersion, teleseismic traveltime and normal-mode splitting function measurements. *Geophys. J. Int.* **184**, 1223–1236, DOI: 10.1111/j.1365-246X.2010.04884.x (2011).

13. Li, M., Wolf, J., Garnero, E. & Long, M. D. Flow and Deformation in Earth's Deepest Mantle: Insights From Geodynamic Modeling and Comparisons With Seismic Observations. *J. Geophys. Res. Solid Earth* **129**, e2024JB029058, DOI: 10.1029/2024JB029058 (2024).
